# The role of Notch signaling in gastric carcinoma: molecular pathogenesis and novel therapeutic targets

**DOI:** 10.18632/oncotarget.17809

**Published:** 2017-05-11

**Authors:** Yizheng Yao, Ying Ni, Jiawen Zhang, Hua Wang, Shihe Shao

**Affiliations:** ^1^ Institute of Laboratory Medicine, School of medicine, Jiangsu University, Zhenjiang, China

**Keywords:** Notch signaling, gastric cancer, stemness, therapeutic targets

## Abstract

Notch signaling, an evolutionarily conserved signaling cascade system, is involved in promoting the progression of different types of cancers. Within the past decades, the Notch signaling pathway has increasingly been shown to have a primary role in deciding the fate of cancer cells and cancer stem cells in the stomach. Most components of Notch signaling are strongly expressed at different levels in gastric carcinoma tissue samples and are associated with a considerable number of clinical parameters. Moreover, crosstalk signaling between the Notch pathway and the Wnt, Ras, and NF-κB pathways promotes the process of gastric carcinogenesis. Consequently, this increases proliferation and prevents apoptosis in gastric cancer cells, and it contributes to the induction of angiogenesis and accelerates the progression of the epithelial-to-mesenchymal transition. Although the Notch signaling pathway presents novel therapeutic targets for cancer therapeutic intervention, there is still a dearth of in-depth understanding of the molecular mechanisms of Notch signaling in gastric carcinoma. In this review, we summarize the landscape of the Notch signaling pathway and recent findings on Notch signaling in gastric cancer. Furthermore, advanced studies and clinical treatments targeting the Notch signaling pathway arediscussed.

## INTRODUCTION

Despite the declining incidence of gastric carcinoma, it remains one of the most common malignancies and the second largest cause of cancer-related deaths in the world [1, 2]. According to epidemiological surveys, the cause of gastric carcinoma includes *Helicobacter pylori* infection, diet, lifestyle, host genotype, and smoking [3, 4]. The development of gastric cancer involves complicated steps and multiple agents, in which *Helicobacter pylori* (*H. pylori*) has a vital role in the process of disease development. Additionally, accumulation of multiple genetic changes, such as gene mutations and chromosome translocations, activate oncogenes and/or repress tumor suppressors and trigger normal cells to become tumor cells [5, 6]. Additionally, epigenetic modification (methylation and acetylation) and miRNA regulation are involved in many types of cancer [7, 8]. In addition, a large number of studies have demonstrated that aberrant expression of signaling pathways may play a direct or indirect role in regulating tumor-related genes and stimulate gastric cancer through complex processes and interactions [9].

The term Notch was first used to describe a morphology phenotype shown as ‘notches’ at the wing margin of *Drosophila* [10]. Thereafter, the Notch sequence of *Drosophila melanogaster* was amplified in 1985 [11]. Notch signaling is a signaling cascade that is evolutionarily conserved and controls many cellular processes, including cell fate determination, cell differentiation, proliferation, tumor angiogenesis, stemness maintenance and apoptosis, which are mediated via cell-to-cell contact and crosstalk with other signaling pathways [12]. The Notch family are transmembrane proteins that function in regulating membrane proteins and nuclear transcriptional agents. It has been demonstrated that there are four Notch receptors (Notch1–Notch4) and five DSL ligands (Jagged1, Jagged2, Dll1, Dll3, and Dll4) in mammals. Notch signaling is initially activated by binding of ligand and receptor on neighboring cells. After two successive proteolytic cleavages, mediated by ADAM/TACE at the extracellular domain and the γ-secretase complex at the transmembrane region, the Notch intracellular domain (NICD) is released into the cytoplasm. It then translocates into the cytoblast and combines with the transcriptional repressor C-promoter binding factor-1(CBF1 in human also know as CSL) to replace a co-repressor complex. Finally, the CSL complex targets and stimulates effector genes such as genes in the *Hes* and *Hey* subfamilies [13–15]. Furthermore, these key Notch pathway effectors are highly expressed in gastric cancer tissues compared with adjacent normal gastric epithelium and are correlated with poor prognosis of patients [16].

This paper presents data regarding the expression level of Notch signaling components and explores the pathogenic role of Notch signaling in gastric tissues. Additionally, based on studies and clinical trials, the three major approaches to induce inhibition of the Notch pathway are highlighted [17], including a) blocking a combination of receptors and ligands; b) inhibiting NICD production; and c) targeting the co-activator complex. The Notch pathway may therefore provide particular targets for gastric cancer prevention, which may be an exciting direction for gastric carcinoma treatment.

## OVERVIEW OF THE NOTCH SIGNALING CASCADE

### Ligands and receptors of the Notch cascade

Based on the structural homology of Delta and Serrate ligands in Drosophila, the Notch ligands in mammals are referred to as Delta-like ligands (Dll1, Dll3 and Dll4) and Serrate-like ligands (Jagged1 and Jagged2) [20], which are type I transmembrane proteins. The intracellular region of the Notch ligands has a chain of 100–150 amino acids in the cytoplasm with no highly homologous sequences [18]. They primarily contain lysine residues and C-terminal PDZ motifs (PSD-95/Dlg/ZO-1), which can send an activation signal to ligands and be ubiquitinylated to trigger endocytosis [19]. The extracellular domain of Notch ligands consists of an N-terminal domain (MNLL), a Delta/Serrate Ligand domain (DSL) and Epidermal Growth Factor (EGF) repeats [20]. Ligands with a DSL domain have greater affinity for Notch receptors than the atypical ligands DNER, F3/Contactin and NB-3 without the DSL domain [21].

The four receptors (Notch1, Notch2, Notch3 and Notch4) are all type I transmembrane proteins with an extracellular domain, transmembrane segment and an intracellular region. The Notch extracellular domain (NECD), with a variable number of EGF-like repeats, three cysteine-rich tandem Lin12/Notch repeat (LNR) domains and a heterodimeric region, can couple to the DSL domain of Notch ligands and activate the signaling cascade [19, 20, 22]. The Notch intracellular domain (NICD) includes a cytoplasmic RAM23 domain, six Ankyrin repeats, two nuclear localization signals, one transcription-activating domain and a PEST domain. Receptors with different domains have different functional purposes, for instance, CSL binds to the RAM domain, Notch regulation agents bind to the ANK repeats, and the PEST region is associated with degradation and the stability of Notch receptors [23].

### Activation of the Notch signaling pathway

**Figure 1:**
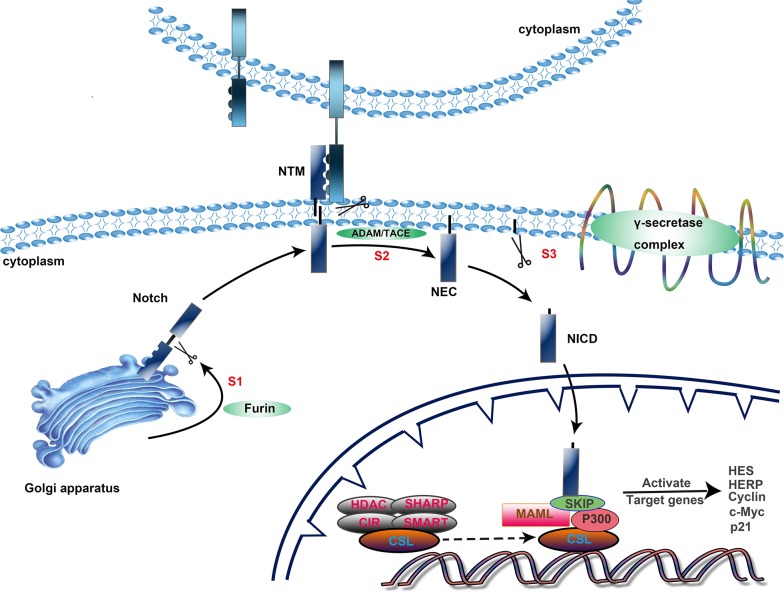
Activation of the Notch signaling pathway The Notch signaling cascade primarily consists of three steps. 1) Intracellular synthesis. The precursor of the Notch receptor becomes a heterodimeric receptor after S1 cleavage by furin-like convertases within the trans-Golgi network and then translocates to the cytomembrane. 2) Proteolysis at the transmembrane region. After two proteolytic cleavages, mediated by ADAM/TACE at the extracellular domain and the γ-secretase complex at the transmembrane region, the NICD is released and transported into the nucleus. 3) Effective stage. The NICD/CSL complex recruits co-activators, including mastermind-like (MAML) and p300, that can accelerate the activation of Notch target genes.

The Notch signaling pathway primarily consists of three proteolytic cleavage phases and endocytosis of the receptors to promote the Notch signaling cascade (Figure 1). First, the 300 kDa precursor of the Notch receptor is cleaved at site 1 (S1) by furin-like convertases within the trans-Golgi network [23]. This produces a heterodimeric receptor consisting of a C-terminal fragment membrane-tethered intracellular domain (NTM) and an N-terminus N(EC) that contains most of the extracellular region, which is then transported to the cytomembrane, followed by ligand-receptor binding and endocytosis [24]. This leads to a conformational transition of the receptor, which exposes two receptor cleavage sites [25, 26]. S2 cleavage by metalloproteases of the ADAM family in the extracellular region of the receptor produces membrane-anchored Notch extracellular truncation (NEXT) [27]. This makes the NEXT susceptible to the next proteolysis at the transmembrane region (S3) mediated by the γ-secretase complex, which includes presenilin1 and 2, nicastrin, Pen-2, and Aph1 [28]. Subsequently, the Notch intracellular domain (NICD) is released and then enters the nucleus. The NICD couples to CSL to replace the co-repressor complex, and thereupon, the co-repressors (HDAC, SHARP, CIR and SMRT) are released. Upon assembling with the NICD, the CSL complex becomes activated to regulate various biological functions. Additionally, the complex recruits co-activators, including mastermind-like (MAML) and p300, that can accelerate the activation of Notch target genes [29, 30]. Among the best-known target genes are two families of transcriptional factors, the Hairy enhancer of split genes (*Hes*) and the Hes-related repressor protein (*HERP*) family. There are also some additional target genes, such as *cyclin D1* [31], *p21* [32], *NF-κB* [33], and *c-Myc* [34].

On the other hand, it has also been reported that the activated NICD of the Notch1 receptor can interact with p50/c-Rel to upregulate the expression of interferon-γ in peripheral T cells by activating NF-κB [35]. In addition, F3/contactin has been discovered to be an original ligand of Notch, specifically initiating a Notch/Deltex1 signaling pathway and upregulating the myelin-related protein MAG to promote oligodendrocyte maturation and myelination [36, 37].

### The notch signaling network

#### Wnt signaling pathway

The Wnt signaling pathway is also a largely conserved signaling system that regulates biological characteristics such as differentiation, proliferation, migration and adhesion, and it especially plays a pivotal role in embryonic development, stem cell differentiation, and tumor growth through complex mechanisms [38]. Several studies have illustrated that these two signaling pathways may have crosstalk through the following mechanisms: a) In Schneider 2 cells, two different forms of Notch receptors increase the expression of Dfrizzled 2, patched, shaggy, and hairy, of which Dfz2 and patched genes possess the ability to activate Wnt signaling [39]. b) Dvl (Dishevelled), a type of cytoplasmic phosphoprotein in the Wnt family, can antagonize Notch signaling by binding to the NICD. c) Increased Notch signaling is available to enhance some of the Wnt-induced transformations through measured expression of the Wnt target genes Axin-2 and Lef-1 [40]. d) Activated GSK3beta possibly phosphorylates the NICD in Notch signaling and protects the intracellular domain from proteasomal degradation [41]. e) Notch signaling initiates Wnt signaling by increasing the expression of the Wnt receptor Frizzled, which is controlled by the CSL transcription factor [42].

#### Ras/MAPK

Complicated interactions between Ras and Notch signaling have been explicitly indicated in mediating cellular functions. Ras increases the expression of Delta1 and Notch1 via p38/MAPK kinase [43]. In studies concerning lung carcinomas, the expression of Notch3 was detected in 80 of 207 (39%) excised lung tumors. Interestingly, the expression of Notch3 was positively correlated with EGF receptor expression. One mechanism of ERK regulation by Notch3 is via transcriptional regulation of MKPs and the tyrosine kinase receptor itself [44]. From another point of view, there exists an antagonization between Ras and Notch during tumorigenesis. In NIH3T3 cells, knocking down Notch expression triggered Fgf-induced transformation (which acts in part through Ras), whereas Fgf is capable of antagonizing the expression of Notch signaling. Therefore, in this situation, Notch functions as a tumor repressor [45, 46].

Nf-κb is an intracellular constitutively active form of the Notch1 receptor that matches the function of IκB with specificity for the p50 subunit and possesses the ability to reduce NF-κB activity in the nucleus. Furthermore, analysis of a GST pull-down assay indicated that the N-terminal amino acid sequence of the NICD decreases NF-κB-induced gene expression independent of the ankyrin repeats. In addition, it is a newfound co-action region and is designated as the NF-κB binding domain (NBD) [47, 48].

## EXPRESSION LEVEL OF NOTCH COMPONENTS IN THE STOMACH

### Notch expression in normal gastric tissues

The gastric mucosa tissues are dominated by a great number of zymogenic cells and parietal cells and a limited number of stem cells and progenitor cells, where Notch, especially Notch1 and Notch2, exerts a fundamental role in regulating gastric epithelial cell homeostasis [49, 50]. Notch activation in the stomach converts epithelial cells into stem and/or multipotential progenitors, which leads to the formation of dysplastic adenomas [51]. Furthermore, *in vivo* Notch maintains homeostasis of the stomach epithelial cells by inducing proliferation of LGR5+ antral stem cells [52]. Importantly, it has been determined that Notch1, Notch2, Notch3, Jagged1, Jagged2 and target genes of Notch signaling (*Hes1* and *Hath1*) are expressed in the isthmus of healthy human stomach lining [53–55]. In addition, the results of Sander's research detected the expression of Notch1, Notch2, Jagged1, and Jagged2 in the mucous membrane of the cardia. Specifically, the highest expression is in the basal layer and is most striking for Notch1 and Jagged2 [56].

### Notch expression in gastric carcinoma

The expression pattern of Notch components and their distribution in different parts of the stomach is likely to have diverse roles in gastric cancer (Table 1). The Notch pathway participates in the process of transformation from gastric epithelial cells to gastric pits. Additionally, Notch1, Notch3, Jagged1, Jagged2 and Hes1 are expressed primarily at the isthmus of gastric mucosa, based on immunohistochemical analysis, and are expressed significantly less in normal gastric tissue compared to gastric cancer tissues [55].

**Table 1:** List of function and associated mechanism of Notch ligands and receptors

Molecules	Function or biological correlation	Associated molecular mechanisms	Ref.
Dll1	associated with the diffuse and mixed types of gastric cancer	methylation of Dll1 inhibits activation of Notch1 and the downstream target gene Hes1.	[58]
Dll3	NC*	NC*	
Dll4	promotes proliferation, migration and invasion of gastric cancer cells and tumorigenicity.	upregulation of Dll4 increases expression of MMP-2 and promotes progression of gastric cancer.	[59]
	suppresses angiogenesis in gastric cancer.	the VEGF-Dll4 signaling network controls new blood vessel sprouts.	[60][61]
Jagged1	functions in maintaining the homeostasis of cancer stem cells.	Wnt signaling activation induces self-renewal of stem cells due to Notch signaling activated by Jagged1, a target gene of Wnt/β-catenin signaling.	[62]
	contributes to aggressiveness and metastasis of gastric cancer.	Jagged1 increases the release of N1IC, which binds to the COX-2 promoter and increases COX-2 expression in a CBF1-dependent pathway.	[16]
Jagged2	associated with intestinal/glandular differentiation.	NC*	[55]
Notch1	essential for the maintenance and differentiation of stem-like cells in gastric cancer.	Notch1 upregulates CD133 expression, a stem-like cell marker, in an RBP-Jκ dependent manner.	[63]
	promotes progression of gastric cancer.	Notch1 signaling releases the N1CD, which binds to the COX-2 promoter and upregulates COX-2 expression in a CBF1-dependent pathway.	[16]
	promotes colony formation, migration and invasion of gastric cancer cells.	Notch1 signaling increases Twist promoter activity through STAT3 phosphorylation.	[64]
Notch2	enhances gastric cancer progression.	N2IC, the activated form of Notch2, binds to the COX-2 promoter and induces COX-2 expression in a CBF1-dependent manner.	[65]
	controls stem cell self-renewal.	Notch2 maintains the stemness state of the stem cell population promoted by suppression of miR-205.	[66]
Notch3	associated with intestinal/glandular differentiation.	NC*	[55]
Notch4	promotes gastric cancer growth.	Notch4 activates Wnt1/β-catenin signaling to accelerate gastric cancer growth.	[67]

* Not clear (NC) indicates that the function or mechanism of the corresponding molecule is not clear from present studies.

More studies have explored the role of Notch1 in gastric carcinoma compared to other components of Notch signaling. Compared to the expression in healthy stomach mucosal tissue, Notch1 expression is significantly higher in gastric carcinoma and is also intimately associated with tumor volume, differentiation grade, depth of invasion and vessel invasion, as confirmed by a tissue microarray [57]. More importantly, the 3-year survival rate is dramatically lower in patients with higher expression of Notch1 [57]. A study undertaken by Piazzi and colleagues to assess the role of Notch1 and the corresponding ligand Dll1 in gastric carcinoma shows that Dll1 is not detected in KATOIII, SNU601, SNU719 and AGS cells from eight different types of gastric cancer cell lines [58]. In addition, upregulated expression of Dll1 has a high correlation with the initiation of Notch1 signaling, with an increased expression of Notch1 intracellular domain (NICD) and the downstream target gene Hes1. With samples from 52 patients with gastric carcinoma and 21 healthy controls, it was revealed that the expression of Dll1 is correlated with Hes1 expression. On the other hand, from an ING-GAS mouse model infected with H. pylori, it was revealed that methylation silencing of Dll1 has the ability to control Notch1 activity in gastric cancer [58]. Collectively, the Dll1-Notch1 signaling axis and the target gene Hes1 are suggested to have important roles in gastric cancer.

Furthermore, the expression of Jagged1 is related to invasion of human gastric carcinoma, thereby leading to a low survival rate compared with the absence of Jagged1 [16]. In addition, it is reported that Notch3 and Jagged2 are not only associated with the development of gastric carcinoma but are also involved in intestinal glandular differentiation of gastric carcinoma cells. This may be a favorable prognostic indicator [55].

## ROLE OF NOTCH SIGNALING IN GASTRIC CARCINOMA

There is a strong correlation between the expression level of Notch2 and the development of gastric carcinoma and a reasonable relationship between Notch1 upregulation and intestinal-likephenotypes of gastric lesions [68]. Transfection of pre-microRNA-181c inhibits proliferation of two types of gastric cancer cells (KATO-III and MKN45) by targeting the downstream oncogenic gene Notch4 [69]. Transduction of retroviral vectors for overexpression of the intracellular domain of Notch (ICN) activates Notch1. This subsequently protects BGC-823 cells from TNFα-induced growth suppression and apoptosis, partially by directly reducing cleaved caspase3 or downstream target genes of caspase3 [70]. Conversely, siRNA against Notch1 promotes apoptosis of BGC-823 cells mediated by TNFα, indicating that Notch1 overexpression can reduce apoptosis in a caspase3-dependent manner [70]. Therefore, this provides a method to suppress proliferation or induce apoptosis of gastric cancer cells by targeting different Notch receptors.

Activation of the Notch1-COX-2 and Notch2-COX-2 signaling axes is also a pivotal event in promoting gastric cancer progression partly through Cyclooxygenase-2. Notch1 receptor intracellular domain (N1IC) and N2IC bind to the promoter region of COX-2 and then increase the activation of COX-2 in a CBF1-dependent manner in SC-M1 cells, additionally enhancing the migration and invasion abilities of SC-M1 cells [16, 65]. Reducing the expression of COX-2 with a COX-2 inhibitor (NS-398) or knocking down COX-2 can suppress the ability of N1IC and N2IC to facilitate tumor progression. Additionally, the restraint of tumorigenic power by knocking down Notch2 in SC-M1 cells was abrogated by treatment with exogenously expressed COX-2 or PGE2 (the main enzyme product of COX-2) [65]. Based on previous findings, it is of great importance that the Notch-COX-2 signaling axis participates in controlling the development of gastric carcinoma.

The interaction of Notch, STAT3 and Twist signaling in gastric carcinoma is reported to have an important function in promoting the development of gastric cancer. Notch1 activation enhances Twist expression and phosphorylated STAT3 levels in gastric adenocarcinoma SC-M1, HEK293 and K562 cells. Moreover, the overexpression of the Notch1 receptor intracellular domain (N1IC) elevates gastric cancer progression, including tumor growth, metastasis, migration and invasion, by promoting the intercellular interplay of STAT3 and the Twist promoter [64]. More importantly, using JSI 124 (a STAT inhibitor) or knocking down Twist, can inhibit tumor growth and lung metastasis induced by N1IC in SC-M1 cells of mice. From clinical gastric cancer specimens, the expression of Notch1 and Jagged1 are tightly correlated with the phosphorylation level of STAT3 and the expression level of Twist [64]. Considering that gastric carcinoma progression is promoted by the Notch1-STAT3-Twist signaling axis, this pathway may offer a potential strategy for gastric carcinoma treatment.

An emerging crucial function of Notch signaling is to stimulate gastric cancer stem-like cells (GCSCs). The Notch pathway is also involved in a series of biological characteristics of cancer stem cells, for instance, radiotherapy endurance [71], chemotherapy resistance [72], and epithelial to mesenchymal transition [73]. Recently, it has been reported that Notch1 maintained a cancer stem-like phenotype in diffuse type gastric cancer by directly regulating CD133, a stem-like cell marker [63]. Similarly, using another gastric cancer stem cell marker, CD44, Notch1 and Hes1 were found to be highly expressed in gastric cancer stem-like cells (GCSCs)of MKN45 cells [74, 75]. Interestingly, *p21*, *Myc*, and the downstream target genes of the Notch pathway are also highly expressed in MKN45 cells. This contributes to an improvement in cancer cell proliferation capacity and secretion of VEGF. Thus, based on these results, the Notch1-mediated proliferation of stem cells stimulates tumor angiogenesis. However, Notch signaling may not independently regulate gastric cancer stem cells, where Wnt signaling is also strongly expressed. Coincidentally, β-catenin (a central factor of Wnt signaling) activates Notch signaling by upregulating Jagged1 expression, which is also a reasonable mechanism in the tumorigenic process of intestinal cancer [76]. The Notch signaling pathway is independently capable of blocking differentiation and inducing angiogenesis in tumoral contexts, but facilitating proliferation and maintaining stemness depends on crosstalk with Wnt or other signaling pathways [77].

## TARGETING NOTCH SIGNALING FOR GASTRIC CARCINOMA THERAPY

Deeper insight into the Notch signaling cascade and its crosstalk with different signaling pathways will give us new understanding and identify effective molecular targets that may lead to the design of new therapeutic strategies. Notch signaling in a wide variety of cancers may be interrupted at different levels, including the following three aspects (Table 2).

**Table 2:** Molecular inhibitors targeting Notch signaling

Therapeutic methods	Molecular inhibitors	Targets	Mechanism	Ref
Blockade of receptor and ligand components	HD105	Dll4 and VEGF	HD105, a bispecific antibody, effectively inhibits angiogenesis and tumor growth by specifically blocking the VEGF/VEGFR2 and Dll4/Notch1 signaling pathways.	[60]
	GSK-3α	Notch1/ NICD	GSK-3α binds to Notch1 (three Thr residues: Thr-1851, -2123, and -2125) and negatively regulates the expression of Notch1 and NICD.	[78]
	256A4, 256A8	Notch3	256A4 and 256A8, monoclonal antibodies, inhibit activation of Notch3.	[79]
	miR34	Notch1, Notch2	miR34 is involved in cancer stem cell self-renewal via regulation of downstream targets Notch1/2 and Bcl-2.	[80]
	miR935	Notch1	miR-935 controls proliferation, migration and invasion of gastric cancer cells by downregulating Notch1 expression.	[81]
	miR124	Jagged1	miR124 negatively regulates Notch1 signaling by targeting Jagged1 in gastric cancer cells.	[82]
	miR181c	Notch4	miR181c may be a tumor suppressor by regulating the expression of target gene Notch4 in gastric cancer.	[69]
	Anti-NRR1 and anti-NRR2	Notch1, Notch2	Paralogue-specific antagonists, anti-NRR1 and anti-NRR2, target Notch1 and Notch2, respectively, in the receptornegative regulatory region	[83]
Inhibitor of NICD production	Numb	NICD	Numb, an endocytic protein, represses Notch activity by binding to the NICD.	[84]
	GSIs	γ-secretase	GSIs are a sort of exogenous chemosynthetic inhibitors (such as DAPT, MRK003, RO4929097, LY411575, MK0752, etc.) that block proteolytic cleavage of Notch by targeting γ-secretase and subsequently reduce activation of NICD and downstream effectors.	[85][86, 87][88][89]
	RECK	ADAM	RECK reduces ADAM-mediated Notch1 shedding and activation by interacting with ADAM10 and ADAM17. It also inhibits gastric stem-like gene expression and sphere formation.	[90]
	miR338-3p	ADAM17	miR338-3p inhibits proliferation, migration and invasion of gastric cancer cells by decreasing ADAM17.	[91]
Target co-activator complex	DN-MAML1	NICD/CSL complex	DN-MAML1 impairs recruitment of MAML1 to combine with the NICD/CSL complex and inactivates downstream target genes.	[92]
	miR199b-5p	Hes1	Blockade of the effector gene Hes1 by miR199b-5p inhibits proliferation of cancer cells and reduces tumor stem-cell populations.	[93]

### Blockade of receptor and ligand components in Notch signaling

Components of the Notch signaling cascade are aberrantly expressed in various types of tumors and tumor-derived cells. An effective way to block Notch signaling is to interfere with receptor-ligand binding. Monoclonal antibodies that specifically bind to Dll4 have been revealed to block Notch signaling in endothelial cells, which inhibits differentiation [94]. Moreover, a Dll4-specific antibody has been shown to inhibit angiogenesis and growth of cancer cells by reducing activation of Notch signaling [95]. One study reported that HNSCC cells, a cell line with high Jagged1 expression, exhibited greatly improved vasculogenesis and tumor growth *in vivo* [46]. Targeting Jagged1 or Dll4 in Notch signaling in human cancers may thus be a promising anti-angiogenic therapeutic approach [96].

MicroRNAs (miRNAs) are approximately 18-22 nt long noncoding RNAs that can modulate protein-coding genes at the post-transcriptional level and have been found to have a significant role in deciding cell fate [97]. Several miRNAs manipulate Notch signaling by combining with the 3′-UTR of protein-coding transcripts of the Notch system. miR124 is reported to inhibit cell growth, migration and invasion of gastric cancer cells and interfere with the cell cycle by targeting Jagged1 and thus negatively regulating Notch1 signaling [82]. miR935 inhibits gastric carcinoma cell proliferation, migration and invasion by targeting Notch1 [81]. The rapid development of RNA interference or other genome-editing techniques thus offers unprecedented prospects for developing anticancer therapeutic interventions [98].

### Inhibitors of NICD production

The interaction between the ligand and receptor promotes cleavage of the Notch receptor at the cell membrane, which is performed by ADAM17 at S2 and the γ-secretase complex at S3, resulting in the cytoplasmic release of the NICD. Therefore, an efficient strategy for Notch-targeted therapy is to inhibit trans-membranous proteolytic cleavage of the Notch receptor.

ADAM17 (also known as TACE) is a transmembrane metalloprotease that plays an important role in the Notch system by cleaving the Notch receptor after ligand binding [27, 99]. Further, expression of ADAM17 has been found to be markedly upregulated in gastric cancer [100]. Hong reported [90] that proteolysis and initiation of Notch receptor signaling induced by ADAM17 are effectively reduced by RECK, which further inhibits the expression of the stemness marker gene CD133 and represses stem cell-like characteristics in GC cells. Downregulation of ADAM17 expression by forced miR338-3p targeting of ADAM17 *in vitro* inhibited proliferation, migration and invasion of GC cells [91]. Taken together, this study provided novel insight and indicated that ADAM17 may be a target for repressing tumorigenesis.

A chemosynthetic γ-secretase inhibitor (GSI), dibenzazepine (DBZ), results in the transformation of proliferative crypt cells into post-mitotic goblet cells in mice carrying a mutation in the tumor suppressor gene Apc, indicating that GSIs can have anti-cancer benefits in colorectal cancer [101]. GSI treatment leads to a significant reduction in the activity of γ-secretase and inhibits activation of the Notch system by restraining the cell cycle at G2/M but with unchanged expression of the γ-secretase complex. This subsequently facilitates cell apoptosis [102]. In addition, treating D283 medulloblastoma xenografts in mice with the dipeptide DAPT, a GSI, results in inhibiting cell proliferation and promoting apoptosis, suggesting that human medulloblastoma proliferation and survival is attributed to Notch activation [103]. Inhibition of Notch signaling through treatment with GSIs prevents BM-mediated drug resistance and makes myeloma cells sensitive to chemotherapeutic agents such as doxorubicin and melphalan, showing an effective approach for the treatment of multiple myeloma [72]. RO4929097 is a GSI that has recently been applied in the clinic. It has been demonstrated to have potent and selective inhibition of γ-secretase, generating an inhibitory effect on the Notch pathway in cancer cells. This was determined by western blotting that showed a reduction in Notch expression and a subsequent downregulation of the target gene Hes1. RO4929097 was not able to inhibit cancer cell proliferation or promote apoptosis; however, it led to a phenotype with more normal properties [87].

Numb was initially discovered as the first intrinsic molecular determinant of cellular processes in *Drosophila* sensory precursor development by asymmetrically partitioning at mitosis. However, recently, it has been identified as a vital repressor in cancers [104]. A primary function of Numb is its involvement in cellular endocytosis, which is critical for NICD release [105]. Moreover, it is reported that over 50% of breast cancers express low levels of Numb, which is correlated with high-grade breast cancers [106]. Interestingly, in response to stimuli from TNFα, IKKα interacts and phosphorylates FOXA2 at S107/ S111, subsequently inhibiting the function of FOXA2. This leads to decreased Numb expression, which then initiates downstream Notch signaling and facilitates cell proliferation and tumorigenesis [107]. Hence, overexpression of Numb may have a unique application potential for gastric carcinoma.

### Targeting the co-activator complex

The NICD, released after Notch receptor-ligand binding, is translocated to the nucleus and forms a complex with CSL, which then boosts recruitment of co-activators in the Mastermind family (MAML1, MAML2, MAML3) [108]. This process finally leads to activation of target genes of CSL, for instance, members of the *Hes* and *Hey* subfamilies [109, 110]. Therefore, inhibiting the co-activators and target genes of the Notch signaling pathway will provide a novel approach for treating stomach cancer with more specific and efficient drugs.

CSL (also known as RBP-Jk or CBF1), is a central node in the signaling cascade for all four Notch receptors (Notch 1–4) [111]. MAML has been revealed to function as a co-activator for effector genes [112]. According to research by Wu and colleagues, various types of cell lines with different levels of MAML expression and activity exhibit varying ability to regulate cell fate through Notch signaling [108]. Additionally, a peptide designated DN-MAML1 has been found to abrogate Notch signaling and control proliferation of T-ALL cells [92, 113]. The tertiary structure of the DN-MAML1 peptide consists of an α-helix, which can couple to the groove of the NICD/CSL complex [114, 115]. This suggests the useful way this peptide blocks recruitment of MAML1 and its combination with the complex, thus directly stopping activation of downstream effector genes [116].

*Hes1* is the most frequently activated target gene of Notch signaling, and it also has a key function in deciding a wide variety of cellular processes [117]. Importantly, Hes1 has been implicated in cycle arrest of hematopoietic stem and progenitor cells [118, 119]. As mentioned above, Notch signaling contributes to maintaining gastric cancer stem cells, which may be associated with Hes1 expression. It is widely acknowledged that CD133 is an indicator of cancer stem cells [120]. Hes1 expression in glioma stem cells is quite high. Moreover, the proliferation of CD133+ glioma stem cells measured by clone formation is significantly inhibited after transfection with shRNA targeting Hes1 [121]. In addition, once the expression of Hes1 is absent, CD133+ cells are undetectable [122]. The same results can be observed in colon and pancreatic cancers [123, 124]. Based on these studies, inhibition of Hes1 may be a promising way to attenuate stemness of gastric cancer cells.

Taken together, we further compiled a list of inhibitors that can be applied to inhibit gastric cancer (Table 3) and dissected their restraint activities. Although Notch signaling in the stomach has garnered much attention, certain issues still need to be addressed. First, a few molecular inhibitors of Notch signaling are not specific only to Notch. For example, GSK-3 kinases, such as GSK-3a, have a broad range of protein targets, only one of which is Notch1. Additionally, miR338-3p targets SOX4 in addition to ADAM17 [127]. Second, a major drawback arising from the use of GSI compounds as a therapeutic is intestinal toxicity complications.

**Table 3:** Molecular inhibitors applied in gastric cancer

Inhibitor	source	category	affected cell lines	functional changes	Ref
HD105	exogenous	Antibody	SCH, SNU-16	increases apoptosis of tumor cells	[60]
				inhibits tumor angiogenesis	
				suppresses tumor progression	
miR124	endogenous	non-coding RNA	SGC-7901, BGC-823	inhibits tumor cell growth	[82]
				suppresses tumor cell migration	
				induces cell cycle arrest	
miR181c	endogenous	non-coding RNA	KATO-III, MKN45	reduces tumor cell proliferation	[69]
miR935	endogenous	non-coding RNA	KATO-III, MKN45	reduces tumor cell proliferation	[81]
				suppresses tumor cell migration	
				inhibits tumor cell invasion	
DAPT	exogenous	chemical synthetic drugs	AGS, MKN45, CD44+ MKN45	inhibits tumor cell growth	[125,126]
				suppresses tumor cell migration	
				inhibits tumor cell invasion	
				prevents epithelial-mesenchymal transition in tumor cells	
				attenuates cancer stem cell renewal	
RECK	endogenous	human gene	GI2, CD133+ MKN45	suppresses sphere formation and growth	[90]
				attenuates cancer stem cell renewal	
miR338-3p	endogenous	non-coding RNA	BGC-823	reduces tumor cell proliferation	[127]
				suppresses tumor cell migration	
				inhibits tumor cell invasion	

## CONCLUSION

Since “Notch” was discovered, nearly one hundred years and numerous studies have revealed the pathogenic mechanisms of Notch signaling in different contexts. Although inhibition of Notch activity is incapable of fully suppressing the effects of gastric cancer, expression of Notch components and the activated Notch system still reflect a potentially serious risk of GC [68]. Notwithstanding the great progress that has been made in targeting Notch signaling for cancer therapy and several types of anti-cancer drugs that have been put into clinical application, there remains a search for more effective and specific Notch-associated anti-cancer strategies: i) Prevention of side effects and drug resistance. According to clinical studies, GSIs are easy for patients to administer and are inexpensive, but we cannot afford to ignore the side effects of treatment with GSIs, such as nausea, emesis, and intestinal epithelial erosion. Worse still is that with long-term use of GSIs, host defense responses develop resistance to GSIs (MRK-003). Thus, it is essential to seek a feasible drug combination, which should avoid unintended effects and overcome drug resistance. ii) Eliminate gastric cancer stem cells. Due to the great importance of GCSCs in cancer initiation, recurrence, drug resistance, and metastasis, as well as a key function of Notch in controlling GCSCs, there is a pressing need to develop an effective medication for anti-gastric cancer by targeting Notch. iii) Greater specificity. GSIs have a broad range of targets in addition to Notch, which indicates non-selective inhibition of several signaling pathways. Perhaps, that is why serious side effects occur. The development of miRNA silencing and polypeptide drugs may provide a precise therapeutic method to target dominant effector genes.

To sum up, Notch signaling acts an oncogenic booster with several therapeutic target sites in gastric cancer. Further *in vitro* and *in vivo* studies are still required to verify whether strategies of targeting the Notch cascade can be applied to clinical treatment with fewer side effects and drug resistance and greater specificity.
